# Enhanced DeepLabV3+ with OBIA and Lightweight Attention for Accurate and Efficient Tree Species Classification in UAV Images

**DOI:** 10.3390/s25247501

**Published:** 2025-12-10

**Authors:** Xue Cheng, Jianjun Chen, Junji Li, Jiayuan Yin, Qingmin Cheng, Zizhen Chen, Xinhong Li, Haotian You, Xiaowen Han, Guoqing Zhou

**Affiliations:** 1 College of Geomatics and Geoinformation, Guilin University of Technology, Guilin 541004, China; cx12@glut.edu.cn (X.C.); lijunji@glut.edu.cn (J.L.); yinjy@glut.edu.cn (J.Y.); chengqingmin123456@glut.edu.cn (Q.C.); czz@glut.edu.cn (Z.C.); lxh@glut.edu.cn (X.L.); youht@glut.edu.cn (H.Y.); xwhan@glut.edu.cn (X.H.); gzhou@glut.edu.cn (G.Z.); 2 Guangxi Key Laboratory of Spatial Information and Geomatics, Guilin University of Technology, Guilin 541004, China

**Keywords:** tree species classification, UAV, object-based image analysis, lightweight attention mechanism, DeepLabV3+

## Abstract

Accurate tree species classification using high-resolution unmanned aerial vehicle (UAV) images is crucial for forest carbon cycle research, biodiversity conservation, and sustainable management. However, challenges persist due to high interspecies feature similarity, complex canopy boundaries, and computational demands. To address these, we propose an enhanced DeepLabV3+ model integrating Object-Based Image Analysis (OBIA) and a lightweight attention mechanism. First, an OBIA-based multiscale segmentation algorithm optimizes object boundaries. Key discriminative features, including spectral, positional, and vegetation indices, are then identified using Recursive Feature Elimination with Cross-Validation (RFECV). High-precision training labels are efficiently constructed by combining Random Forest classification with visual interpretation (RFVI). The DeepLabV3+ model is augmented with a lightweight attention module to focus on critical regions while significantly reducing model parameters. Evaluations demonstrate that the improved DeepLabV3+ model achieved overall accuracy (OA) of 94.91% and Kappa coefficient (Kappa) of 92.89%, representing improvements of 2.91% and 4.11% over the original DeepLabV3+ model, while reducing parameters to 5.91 M (78.35% reduction). It significantly outperformed U-Net, PSPNet, and the original DeepLabV3+. This study provides a high-accuracy yet lightweight solution for automated tree species mapping, offering vital technical support for forest carbon sink monitoring and ecological management.

## 1. Introduction

Forest ecosystems, as a core component of terrestrial ecosystems, serve vital ecological functions including carbon sink sequestration, soil-water conservation, and biodiversity conservation [1]. The composition and distribution of tree species directly affect the accuracy of forest carbon storage estimation and ecosystem stability assessments [2]. However, accelerating urbanization and intensified human activities are degrading forest ecosystem structures and functions [3], particularly in ecologically vulnerable regions such as karst areas, where forest stand fragmentation has increased markedly [4], triggering progressive decline in ecological services. Therefore, acquiring accurate information on tree species has become a core scientific issue for coordinating ecological red line management and sustainable resource development.

In recent years, the rapid development of unmanned aerial vehicle (UAV) remote sensing technology has provided new technical means for dynamic monitoring and precise management of forest resources [5]. However, while its high-resolution feature enhances the observation accuracy, it also brings significant technical challenges. In tree species classification, spectral similarity and canopy heterogeneity diminish the precision of traditional classification methods [6], and pixel-based segmentation methods are prone to generating “salt-and-pepper noise” [7]. Although deep learning can effectively extract features, its performance is highly dependent on the quality of training labels. When the annotation errors of training samples exceed a certain proportion, the overall accuracy (OA) of deep learning models declines [8]. Although the traditional manual visual interpretation method can maintain high annotation accuracy, it needs professional interpreters to devote considerable time to meticulous annotation, thereby creating a severe efficiency bottleneck in large-scale regional applications. In addition, due to differences in the judgment criteria of key features such as crown boundaries among different interpreters, the consistency of annotation results is difficult to guarantee. Therefore, manual labeling and low standardization have become a bottleneck restricting the large-scale application of deep learning technology in the field of forest remote sensing.

Consequently, there is an urgent need for an integrated framework that can simultaneously address the challenges of efficient label generation and accurate, lightweight model inference in complex forest environments. This study takes the typical karst forest area of the Lijiang River Basin as the research area. Based on high-resolution UAV images, it combines the Object-Based Image Analysis (OBIA) method and an improved DeepLabV3+ model for tree species classification. The main research objectives are as follows: (1) Optimize the image segmentation scale and screen key features through multiscale segmentation algorithms and feature selection methods. (2) Construct high-precision training labels by integrating the Random Forest classifier with the visual interpretation (RFVI) method to improve label production efficiency. (3) Introduce lightweight networks and attention mechanisms to modify the DeepLabV3+ model, enhancing its segmentation performance and computational efficiency.

The remainder of this paper is organized as follows: Section 2 reviews related work; Section 3 describes the research data and methods; Section 4 presents the experimental results; Section 5 provides a discussion; and Section 6 concludes the paper.

## 2. Related Works

### 2.1. Traditional Classification Methods and OBIA

To tackle the technical bottlenecks of tree species classification in high-resolution UAV images, researchers have pioneered a plethora of innovative approaches [9,10,11]. Among traditional methods, the Object-Based Image Analysis (OBIA) method has been widely adopted. It harnesses multiscale segmentation to synergize spectral, textual, and spatial features, thereby substantially augmenting classification precision [12]. However, despite its effectiveness in reducing salt-and-pepper noise, OBIA typically relies on shallow machine learning classifiers and often encounters computational inefficiencies and complex feature engineering processes when processing complex forest landscapes.

### 2.2. Deep Learning in Semantic Segmentation

In contrast to traditional methods, deep learning models have revolutionized remote sensing image classification with their robust automatic feature extraction capabilities [13,14]. Yet, early models were often plagued by limitations such as constrained local receptive fields and suboptimal utilization of feature channels. In response, researchers have successively devised multiscale feature fusion architectures. Notably, the DeepLabV3+ model excels in capturing fine-grained image details and boosting classification accuracy by employing dilated convolution to expand the receptive field, leveraging spatial pyramid pooling for multiscale feature integration, and utilizing an encoder–decoder framework to preserve spatial information [15,16]. Nevertheless, standard DeepLabV3+ networks typically possess a large number of parameters, making them computationally expensive and difficult to deploy for efficient, large-scale forestry tasks.

### 2.3. Attention Mechanisms and Lightweight Networks

To further optimize model performance and efficiency, recent studies have focused on attention mechanism enhancement strategies [17,18,19]. For instance, channel attention modules (CAM) [20,21] enhance discrimination among tree species with similar spectral signatures through feature channel recalibration, while spatial attention modules (SAM) [22,23] refine the identification of crown boundaries in complex terrains. Despite these advancements, current techniques still grapple with a critical trade-off: achieving high accuracy often comes at the cost of excessive computational resource demands. This underscores the research gap addressed in this study: the development of a lightweight yet accurate solution that integrates the strengths of efficient OBIA-based labeling and attention-enhanced deep learning.

## 3. Materials and Methods

### 3.1. Study Area

**Figure 1:**
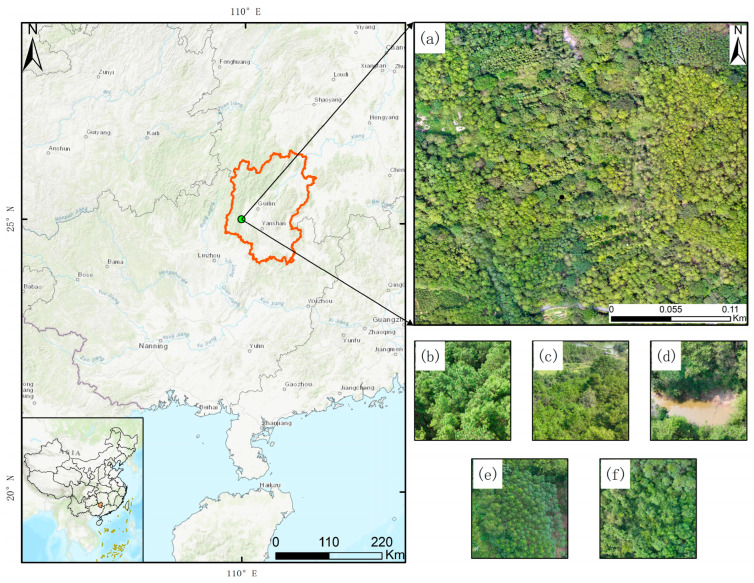
Overview of the study area. (**a**) Drone image of the complete study area, (**b**) Masson pine, (**c**) mixed forest, (**d**) others, (**e**) camphor trees, (**f**) coniferous forest.

The Lijiang River Basin (24°55′–25°50′ N, 110°10′–110°45′ E), situated in Guilin City, Guangxi Zhuang Autonomous Region, China (Figure 1), is an important tributary of the Pearl River Basin, with a total length of approximately 164 km and a drainage area of 1290 km^2^ [24]. The region is renowned for its typical karst peak forest landform and belongs to the subtropical monsoon climate, with an average annual temperature of 19.3 °C and annual precipitation of 1,900 mm. The basin features high forest coverage, dominated by evergreen broad-leaved and bamboo forests, boasting rich biodiversity and significant carbon sink potential [25]. As a national-level scenic area and key ecological function zone, the Lijiang River not only provides the main water source for Guilin City, but its unique ecosystem is also crucial for maintaining regional ecological balance. In recent years, with the intensification of human activities such as tourism development, the Lijiang River Basin has faced threats of forest degradation and declining ecological functions [26]. To support regional ecological protection and carbon sink management, this study selects a typical 300 m × 300 m plot within the basin for the high-precision tree species classification research. This plot encompasses the main vegetation types of the basin, providing an ideal research scenario for UAV remote sensing monitoring.

### 3.2. Data Acquisition and Preprocessing

The UAV data were collected in September 2024 within a study area characterized by diverse tree species, under clear and windless weather conditions. The DJI Mavic 3E UAV, equipped with the O3 Enterprise image transmission system and a 4/3-inch CMOS sensor (weighing 920 g), was employed for data acquisition. Using the DJI RC Pro Enterprise controller, the flight path was meticulously planned and executed in real-time. Flight parameters were set as follows: 80% forward overlap, 70% lateral overlap, a speed of 10 m/s, and a vertically downward-oriented lens, which enabled the capture of RGB images with a 2 cm resolution at an altitude of 118 m. For data pre-processing, Agisoft PhotoScan Professional software V1.4.1 (Agisoft LLC) was utilized to generate digital orthophotomaps (DOMs) through image stitching. Subsequently, coordinate transformation and boundary cropping were performed in ArcGIS 10.8, yielding high-precision mapping products of the study area.

### 3.3. Methods

**Figure 2:**
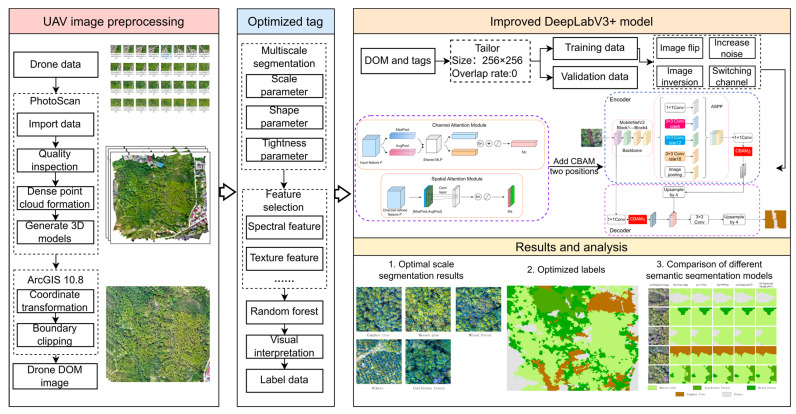
Technical flowchart.

This study constructed a complete technical framework for tree species classification using UAV images, employed a multi-stage processing approach to achieve high-precision classification objectives. Firstly, a full-process data processing from original images to the DOMs was conducted based on the PhotoScan software V1.4.1 (Agisoft LLC) platform. Secondly, a multiscale segmentation algorithm was used to determine the optimal segmentation scale, which was combined with RFECV to identify the optimal feature subset. To enhance the quality of training data, an RFVI method was proposed to establish a high-quality labeled dataset. Finally, the CBAM attention mechanism was integrated into the DeepLabV3+ framework. Through multiscale feature fusion and channel–spatial attention weighting, the model’s feature discrimination ability was enhanced, completing a lightweight model design (Figure 2).

#### 3.3.1. Multiscale Segmentation Algorithm

This study employed a multiscale segmentation algorithm to process UAV images of the study area. Unlike single-scale segmentation, this approach is better suited for analyzing and modeling features in complex scenes. Multiscale segmentation involves adjusting the scale parameters of remote sensing images to achieve hierarchical segmentation [27]. The size of image objects significantly impacts classification accuracy. Three key parameters govern multiscale segmentation: the scale parameter, shape factor, and tightness factor. The shape factor balances the influence of shape and spectral values, while the tightness factor controls the compactness of image objects. Drawing on prior research and iterative experiments, the shape factor and the tightness factor were set at 0.3 and 0.5, respectively, with equal weights (set to 1) assigned to the red, green, and blue bands of UAV images. The scale parameter is central to the multiscale segmentation algorithm. To identify the optimal segmentation scale for the study area, the ESP2 tool developed by Drăguţ [28] was utilized. With a fixed shape factor of 0.3 and a tightness factor of 0.5, ESP2 automatically segments user-defined data by incrementally adjusting the scale parameter and calculating the local variance at each scale to assess the optimal value. Using the ESP2 V2014 scale evaluation software and 13 user-defined parameters, the optimal scale for each object in the study area is automatically determined. By establishing the relationship between segmentation scales, local variance, and the rate of change in local variance (*ROC*-*LV*), the scales corresponding to the peak values of the *ROC*-*LV* curves are selected as the optimal segmentation scale candidates for different objects.

(1) $$ROC=\left[\frac{LV_{\left(L\right)}-LV_{\left(L-1\right)}}{LV_{\left(L-1\right)}}\right]\times 100$$

where *ROC* represents the rate of change in local variance; ${LV}_{\left(L\right)}$ denotes the average standard deviation of the current layer *L*; ${LV}_{\left(L-1\right)}$ denotes the average standard deviation of the previous layer *L* − 1.

#### 3.3.2. Feature Selector

In machine learning and pattern recognition, features represent quantifiable object attributes, and the feature selection process significantly impacts model performance. This study utilized RFECV for feature optimization. By integrating Recursive Feature Elimination with cross-validation [29], RFECV automates feature selection. It iteratively trains the model, successively removing less significant features while using cross-validation to assess the stability of feature subsets, thus automatically determining the optimal feature count [30]. Compared to traditional RFE, RFECV mitigates overfitting risks and improves model generalization. Its key advantage stems from the dual processes of feature ranking and performance evaluation. Cross-validation ensures consistent performance of selected feature subsets across data partitions, reducing biases from single training sets. Appropriate iteration steps and termination criteria were set to balance efficiency and reliability during implementation. To address the high spectral similarity of tree species in the study area’s forestland, a multidimensional feature space framework was constructed. From images segmented at the optimal scale, five major feature categories—spectral, vegetation indices, geometric, positional, and textural features—are systematically extracted, forming the initial feature space. This multi-feature fusion approach integrates multi-dimensional information, enhancing tree species classification accuracy and reliability [31]. Textural features overcome the limitations of spectral data for similar species discrimination, geometric features capture canopy morphological differences, and positional features supply spatial context, all contributing to improved classification. During extraction, the complementarity and synergy among feature types are prioritized to ensure the feature space comprehensively represents research object characteristics. Feature-specific parameters and formulas are detailed in Table 1 and Table 2.

#### 3.3.3. Label Optimization by RFVI Method Combining Random Forest Classification with Visual Interpretation

In deep learning model training, labels are pivotal as they directly affect the model’s generalization capability, performance, and training efficacy. Current research predominantly relies on two approaches: using ArcGIS software to create custom datasets [33] or leveraging public datasets for model training [34,35]. Public datasets, however, suffer from limited variety and narrow applicability. Moreover, due to the unique characteristics of research areas or objectives, many studies require custom datasets. Conversely, creating datasets with ArcGIS software is not without drawbacks—it is vulnerable to labelers’ subjective biases and demands substantial time and labor, which can hinder subsequent deep learning model training. To address these issues, this study integrated the Random Forest classifier with visual interpretation to generate the necessary dataset. This hybrid method not only ensured labeling accuracy but also boosted efficiency, reduced time, and labor costs. Specifically, following multiscale segmentation and feature optimization, the Random Forest classifier was first applied to conduct preliminary classification of UAV remote sensing images across the entire study area. Then, considering the actual conditions of the study area, visual interpretation was used to refine these preliminary results, which ultimately produced a high-precision tree species label map for the study area.

#### 3.3.4. DeepLabV3+ Semantic Segmentation Method

**Figure 3:**
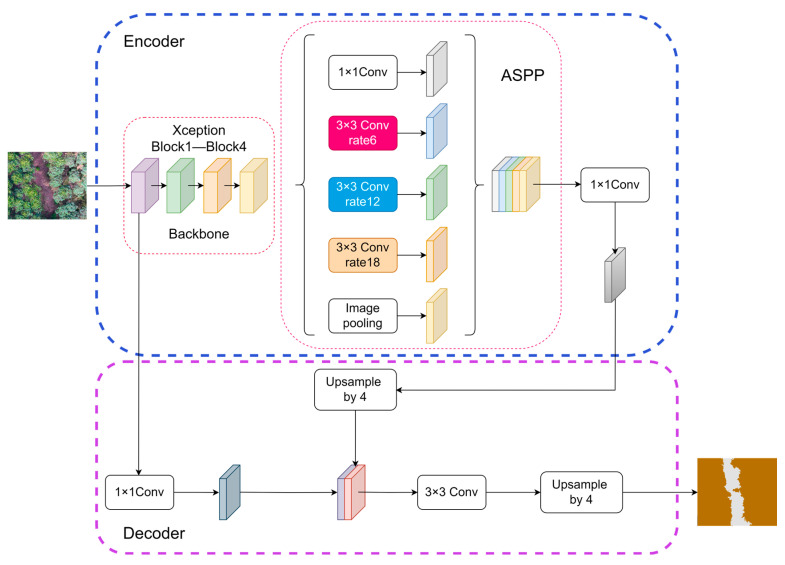
Structure of the DeepLabV3+ model [37].

The DeepLabV3+ model, the latest advancement in the renowned DeepLab algorithm family [36], stands as a cutting-edge convolutional neural network (CNN) for semantic segmentation. As depicted in Figure 3, its architecture consists of an encoder and a decoder. In the encoding stage, dilated convolutions are employed as a pivotal innovation. This technique expands the network’s receptive field without sacrificing original information or escalating computational complexity, enabling the extraction of contextual features from a wider area and enhancing the discriminative ability for various objects and scene elements. Central to its design is the Atrous Spatial Pyramid Pooling (ASPP) module, which utilizes dilated convolutions to effectively integrate multiscale information. By applying distinct dilation rates across parallel convolutional branches, the ASPP module processes features at different scales, thus accommodating objects of varying sizes and hierarchical feature representations. During decoding, DeepLabV3+ refines segmentation boundaries through a unique feature fusion strategy. By merging low-level features—rich in spatial details like textures and edges—with high-level semantic information, the model achieves precise object boundary delineation while maintaining semantic consistency. This approach ensures that the final segmentation results are both semantically accurate and spatially detailed.

#### 3.3.5. Improved DeepLabV3+ Semantic Segmentation Method

**Figure 4:**
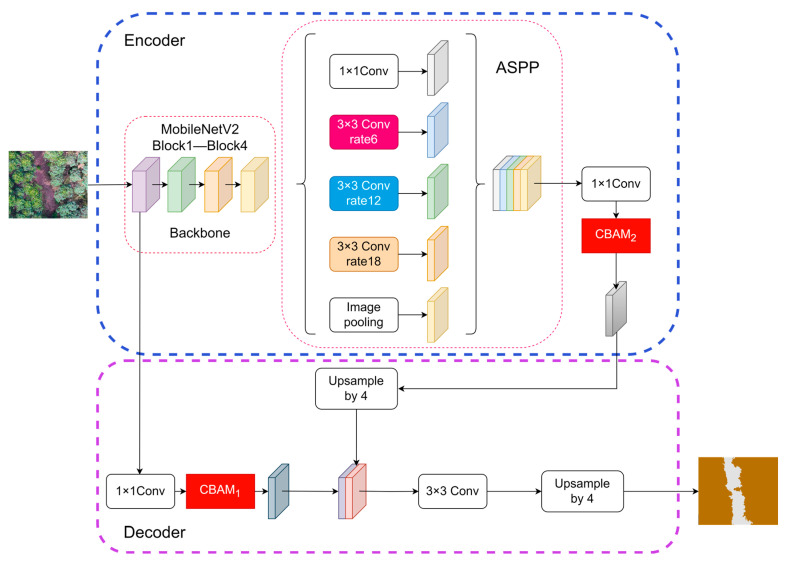
Structure of the improved DeepLabV3+ model.

In response to the complexity of forest tree species and the high resolution of UAV images, the DeepLabV3+ network was modified, with the improved architecture illustrated in Figure 4. In the encoder, the original backbone feature extraction network was replaced by the lightweight MobileNetV2 for feature extraction, which generated both low-level and high-level features. These features were separately transmitted to the decoder and the ASPP module for information extraction and fusion. Meanwhile, the CBAM_1_ attention mechanism was added to the decoder to further integrate the shallow feature extraction capability of MobileNetV2 and enhance the image segmentation performance. Following the ASPP module, the CBAM_2_ attention mechanism was introduced, which weighted the feature maps from ASPP through the collaborative operation of channel and spatial attention. This enhancement boosted feature representation, enabling the network to adaptively focus on critical information (Both CBAM_1_ and CBAM_2_ are instances of the Convolutional Block Attention Module; subscripts are added to distinguish their integration locations).

##### Improvement of the Backbone Network

The original DeepLabV3+ model employs Xception [38] as its backbone network, which integrates residual connections inspired by ResNet [39]. However, Xception has inherent limitations: as a deep neural network with extended depthwise separable convolutions and residual connections, its architecture is complex, featuring a large parameter volume and high computational demands. These characteristics prolong training time and increase resource consumption, which are particularly critical for processing high-resolution UAV forest images—where subtle spectral–spatial differences among land cover types and large data volumes amplify inefficiencies. To address these challenges, this study replaced Xception with the lightweight MobileNetV2 as the backbone network. MobileNetV2 achieves efficiency through depthwise separable convolutions, which decompose standard convolutions into depthwise and pointwise operations. This structural factorization significantly reduces parameters and floating-point operations, enabling the model to maintain high performance while minimizing computational overhead and accelerating inference. These advantages facilitate rapid deployment on resource-constrained devices and align with the real-time processing requirements of UAV remote sensing applications.

##### Introduction of the Attention Mechanism Module

**Figure 5:**
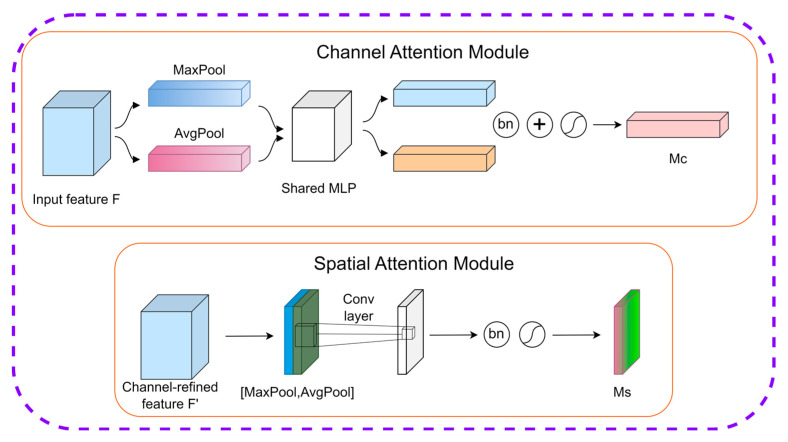
Structure of the CBAM module [41].

In the field of deep learning, a diverse array of attention models exists, each offering distinct approaches to enhancing the performance of CNNs. Among these, the CBAM, proposed by Woo [40] in 2018, has emerged as a prominent attention mechanism, demonstrating unique advantages in the evolution of CNN architectures. Characterized by its simplicity and efficiency, the CBAM serves as a powerful tool for boosting CNNs’ performance, with its overall architecture illustrated in Figure 5.

While attention mechanisms like Squeeze-and-Excitation (SE) [42] and Efficient Channel Attention (ECA) [43] have proven effective in various tasks, they primarily focus on modeling interchannel dependencies. This approach effectively answers ‘what’ features are important, but often neglects ‘where’ these features are located. However, for the task of tree species classification, spatial information is crucial. Tree species are often distinguished not just by general spectral characteristics but by fine-grained spatial details such as texture patterns and canopy shapes. The CBAM is superior in this context because it integrates both the Channel Attention Module (CAM) and the Spatial Attention Module (SAM). Notably, prior research has widely applied the CBAM to the field of forest tree species classification, where it has consistently demonstrated superior performance and robustness [44,45]. Therefore, we selected the CBAM to enhance the feature extraction capability for our specific dataset. Compared with other attention modules, the CBAM features a unique design philosophy: its core lies in sequential information inference and processing across two independent dimensions (channel and spatial), providing an effective strategy for adaptive feature optimization. By deeply mining intrinsic feature information, it enables the network to efficiently extract and utilize critical features. Notably, the CBAM’s lightweight nature endows it with strong versatility and flexibility. It can be seamlessly integrated into various neural network architectures without introducing additional modular overhead, making it highly adaptable across diverse application scenarios to enhance network performance.

In feature processing, the CBAM realizes fine-grained optimization through the sequential application of CAM and SAM. For CAM specifically, global average pooling and max pooling are first applied to the input feature F with size H × W × C to generate two 1 × 1 × C features. These descriptors are processed by a two-layer ReLU-activated neural network, fused via summation, and compressed into the range [0, 1] using the Sigmoid function to obtain the channel weight coefficient Mc. Finally, Mc is multiplied by the original feature F to scale it along the channel dimension, enhancing informative channels while suppressing irrelevant ones. The weight coefficient Mc can be calculated using the following formula:

(2) $$M_{c}(F)=\sigma (MLP(AvgPool(F))+MLP(MaxPool(F)))$$

where *M_c_*(*F*) denotes the channel attention map; *F* ∈ *R*^*H*×*W*×*C*^ represents the input feature map with height *H*, width *W*, and channels *C*; *AvgPool* and *MaxPool*, respectively, represent the global average pooling and max pooling operations; *MLP* represents the multi-layer perceptron, and *σ* represents the Sigmoid activation function.

Similarly, the SAM is critical. For the input feature F′ of size H × W × C, average pooling and max pooling yield two H × W × 1 channel descriptors, which are concatenated along the channel dimension to form an H × W × 2 feature. To extract spatial importance, this concatenated feature is processed by a 7 × 7 convolutional layer with Sigmoid activation, which learns and generates the spatial weight coefficient Ms ∈ [0, 1]. Finally, multiplying Ms by F′ performs spatial feature scaling, highlighting key regions and suppressing insignificant ones to output a spatially optimized feature. The weight coefficient Ms is calculated as follows:

(3) $$M_{s}(F)=\sigma (f^{7\times 7}([AvgPool(F);MaxPool(F)]))$$

where *M_s_* ∈ *R*^*H*×*W*×1^ represents the generated spatial weight coefficient; *F* ∈ *R*^*H*×*W*×*C*^ represents the input feature map with height *H*, width *W*, and channels *C*; *f*^7×7^ represents a 7 × 7 convolution operation, [*AvgPool*(*F*); *MaxPool*(*F*)] indicating the concatenation of the average pooling and max pooling results along the channel axis.

#### 3.3.6. Experimental Environment and Accuracy Evaluation Indicators

This study built a model within the Python 3.9 programming environment and the PyTorch 1.12.1 deep learning framework and completed the computing tasks on the Windows 10 operating system and the NVIDIA GeForce RTX 4070 Ti SUPER (16 GB) graphics card platform. Due to the excessive scale of the original image, to avoid memory overflow and improve computational efficiency, images were cropped into 256 × 256-sized data. The UAV image of the study area was spatially divided into five non-overlapping sub-regions. One sub-region was reserved as the independent test set. The data from the remaining four sub-regions were assigned to the training and validation sets, respectively, with a ratio of 7:3. The cross-entropy loss function (CrossEntropyLoss) and Adam optimizer were used for 100 rounds of iterative training. The initial learning rate was set at 0.001, and a dynamic attenuation strategy was introduced. The batch size was fixed at 8. During training, the model’s generalization performance was enhanced through multi-dimensional data augmentation: (1) Mirrored operations on images and labels along the X-axis and Y-axis, respectively; (2) Randomly swapped among multiple channels of the image, while keep the labels unchanged; (3) Simultaneously performed random rotations of 90°, 180°and 270° on the images and labels; (4) Randomly added Gaussian noise to the image while keep the label unchanged.

For large-scale remote sensing image prediction, a sliding window strategy was employed, dividing images into 256 × 256-pixel patches with 50% overlap. To eliminate edge effects during patch stitching, a central region prioritization mechanism was applied, combined with probability-weighted fusion and morphological post-processing to ensure continuous object boundaries and smooth classification results. A reliable accuracy assessment benchmark was established by generating 550 uniformly distributed vector points across the study area using UAV images with a spatial resolution of 0.02 m. A high-confidence ground truth dataset was developed through manual visual interpretation. The accuracy verification used OA and Kappa to evaluate the classification effect of the entire domain and determined the tree species recognition accuracy through producer accuracy (PA) and user accuracy (UA). The calculation formulas for the four metrics are as follows:

(4) $$OA=\frac{\sum_{i=1}^{K}TP_{i}}{N}\times 100\%$$

(5) $$Kappa=\frac{P_{O}-P_{e}}{1-P_{e}}$$

(6) $$PA_{i}=\frac{TP_{i}}{C_{+i}}\times 100\%$$

(7) $$UA_{i}=\frac{TP_{i}}{C_{i+}}\times 100\%$$

where *TP_i_* represents the number of correctly classified samples of the *i*-th category, *K* is the total number of categories, *N* is the total number of samples, *P_o_* is the actual consistency rate, *P_e_* is the theoretical random consistency rate, *C_i_*_+_ is the predicted total number of the *i*-th category, and *C*_+*i*_ is the true total number of the *i*-th category.

## 4. Results

### 4.1. Multiscale Segmentation of UAV Images

**Figure 6:**
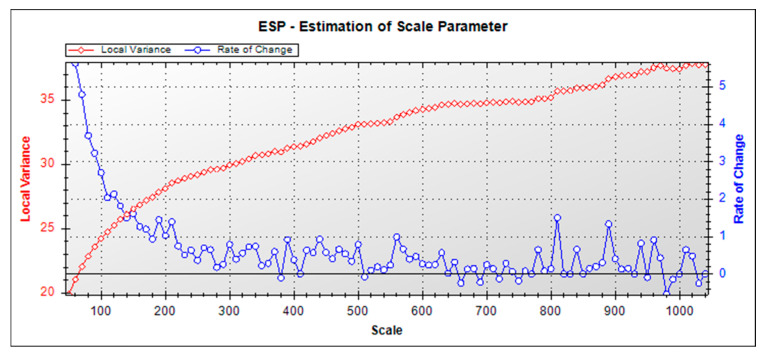
Analysis results of the ESP tool.

**Figure 7:**
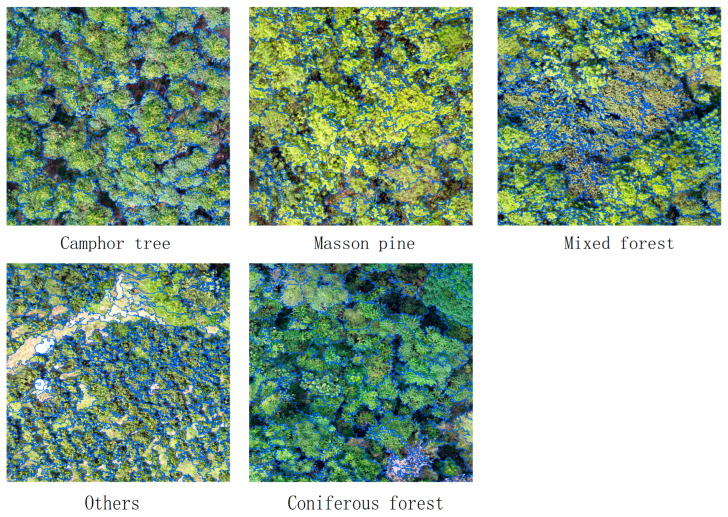
Segmentation results of various tree species. The blue lines are the Image Object Outlines.

Figure 6 presents the *ROC*-*LV* curves of the study area’s remote sensing images generated using the Chart tool in ESP2. The curve peaks occurred at scale parameters 120, 150, 190, 300, 390, and 560. At scales 120 and 150, canopy-layer tree species and other ground features exhibited excessive fragmentation, producing numerous small-segmented objects that hinder classification accuracy. Conversely, scales 300, 390, and 560 resulted in under-segmentation: small-canopy tree species remained unsegmented, mixed-species objects contained multiple tree types, and other features formed overly large homogeneous regions. Optimal segmentation occurred at scale parameter 190, where object sizes aligned with actual ground feature dimensions. Generated segments were neither excessively large nor small, and accurately represented image features. Segmentation boundaries for tree species and other ground features closely matched real-world contours, featuring precise, distinct edges that maintain object integrity (Figure 7).

### 4.2. Feature Optimization

**Figure 8:**
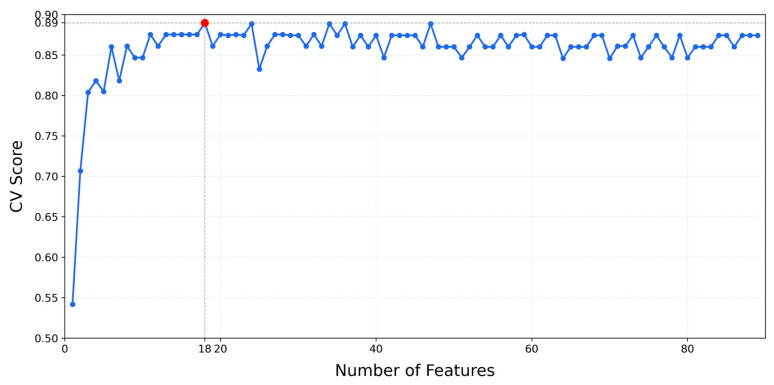
Number of features and cross-validation score. The dotted lines and red dots represent the identification of the “optimal number of features”.

Considering the characteristics of UAV images and the dataset type in the study area, the Random Forest model was selected as the baseline. A five-fold cross-validation was employed to automatically determine the optimal number of features, and the optimal feature set was further validated using the same cross-validation to calculate the average score. Figure 8 depicts the relationship between the number of features and cross-validation scores, showing that the maximum cross-validation score peaks at 0.8923 when the feature count reaches 18, with an average cross-validation score of 0.9038. This demonstrated that the 18 features selected by the Random Forest model were most conducive to improving model accuracy and generalization ability, including spectral features: Red, Green, Blue, Mean_R, Mean_G, Mean_B, Standard deviation B; vegetation indices: RGRI, NGRDI, NGBDI, GLI, and EXG; positional features: X Max, Y Center, X Min, Y Min, X Center, and Y Max.

### 4.3. Label Optimization by RFVI Method Label Optimization

**Figure 9:**
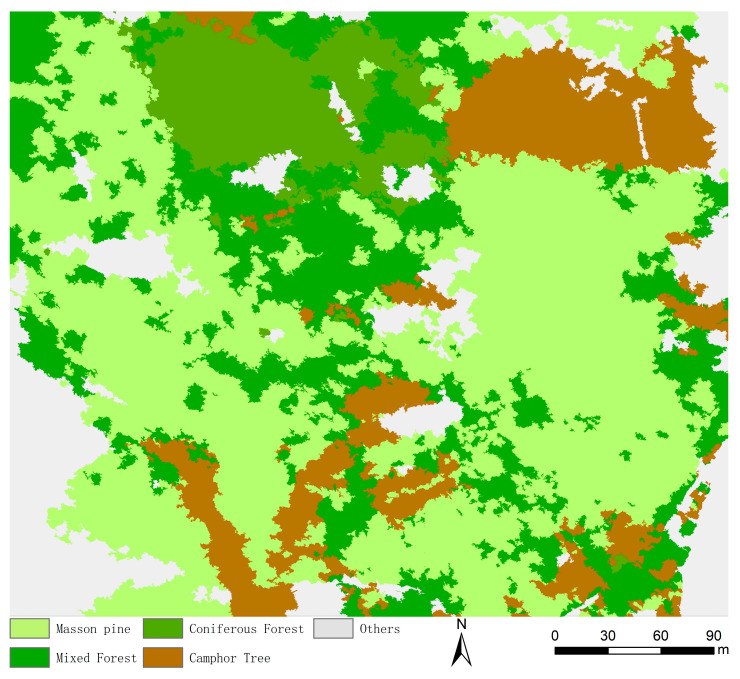
Label optimization results.

The label optimization results obtained after correcting with the combination of the Random Forest classifier and visual interpretation (Figure 9 and Table 3). It can be seen from the figure that the label optimization results were highly consistent with the real ground data. According to the table, the PA of Masson pine is 0.97 and the US is 1, indicating that there was almost no missing classification in this category, and the classification results were completely reliable. The PAs of mixed forest and other categories are both 1, indicating that these two types of ground objects were completely identified in the real samples, while the US were 0.98 and 0.96, respectively, indicating that the classification results were highly credible. The PAs of camphor tree and coniferous forest were both 1, and the UA were 0.99 and 0.98, respectively, further verifying that the model had good classification effects on these two types of ground objects. The OA is 0.9855, and the Kappa is 0.9798, indicating that the model had high global classification accuracy and the classification results were highly consistent with the real distribution. This result showed that the RFVI method performed excellently in the corrected classification task, and the classification results were reliable.

### 4.4. Results of the Improved DeepLabV3+ Model

#### 4.4.1. Ablation Experiment

This study made multiple modifications to the encoder and decoder of the DeepLabV3+ model and conducted accuracy evaluations on them (Table 4).

It demonstrated the impact of different improved modules on model performance, compared to the original model, MobileNetV2, CBAM_1_, CBAM_2,_ and their corresponding OA, Kappa, and parameter counts. The baseline model achieved an OA of 92%, a Kappa of 88.78%, and contained 27.30 M parameters. The original architecture part, replaced with MobileNetV2, reduced the OA and Kappa to 88.73% and 84.31%, respectively, but significantly decreased parameters to 5.81 M, highlighting MobileNetV2′s effectiveness in model lightweighting. CBAM_1_ added to the MobileNetV2-based model boosted the OA and Kappa to 90.73% and 87.01%, with a marginal parameter increase to 5.90 M, indicating that CBAM_1_ improves feature discrimination without substantial overhead. The final model integrated MobileNetV2, CBAM_1_, and CBAM_2_ simultaneously, achieving state-of-the-art performance: an OA of 94.91%, a Kappa of 92.89%, and 5.91 M parameters. Compared to the original model, this represented increases of 2.91 and 4.11 percentage points in the OA and Kappa, respectively, alongside a significant parameter reduction demonstrating concurrent optimization of performance and lightweight architecture.

#### 4.4.2. Experimental Results of Different Semantic Segmentation Models Comparison Results of Different Semantic Segmentation Models

To validate the effectiveness of the proposed algorithm improvements, three widely used semantic segmentation models—U-Net, PSPNet, DeepLabV3+—and the improved DeepLabV3+ were trained and evaluated on the same dataset using identical evaluation metrics (Table 5).

For the OA, PSPNet, DeepLabV3+, and U-Net achieved 90.36%, 92.00%, and 92.91%, respectively, while the improved DeepLabV3+ model achieved 94.91%—significantly outperforming the baseline models and demonstrating its superiority in overall segmentation accuracy. In terms of the Kappa, these models recorded 86.54%, 88.78%, and 90.12%, respectively, with the improved DeepLabV3+ achieving 92.89%—further confirming its advanced classification consistency. Regarding model parameters, PSPNet, DeepLabV3+, and U-Net contained 47.76 M, 27.30 M, and 31.04 M, whereas the improved DeepLabV3+ drastically reduced this to 5.91 M. This reduction demonstrated that the improved model enhanced accuracy while significantly decreasing complexity and improving computational efficiency. For the PA, the improved DeepLabV3+ excelled in categories such as Masson pine (97.56%), mixed forest (93.88%), others (90.22%), camphor tree (93.06%), and coniferous forest (95.24%). It outperformed comparative models in certain classes and matched or surpassed top performers in others, showcasing robust class-specific discriminative ability.

**Figure 10:**
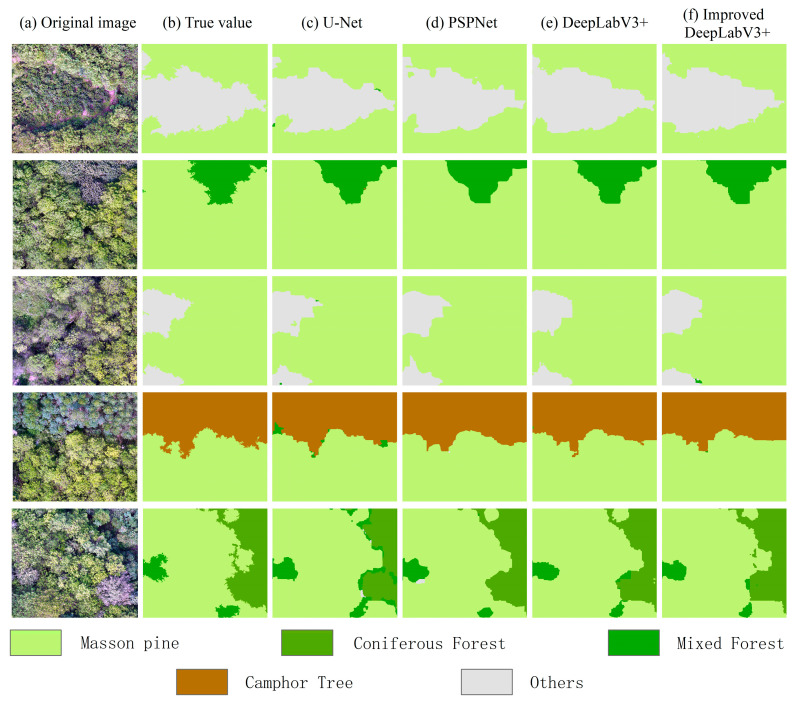
Comparison of segmentation effects of different semantic segmentation models.

Partial segmentation results of PSPNet, DeepLabV3+, U-Net, and the improved DeepLabV3+ semantic segmentation model for the test area are shown in Figure 10. U-Net exhibited blurred boundaries among vegetation types, particularly in Masson pine and mixed forest regions, indicating insufficient detail capture and evident misclassification. PSPNet improved segmentation fidelity but still struggled with precise boundary delineation in camphor tree and coniferous forest areas, showing regional confusion. DeepLabV3+ outperformed the prior models in the OA yet retained minor boundary artifacts in Masson pine and mixed forests, suggesting room for encoder feature extraction enhancement. The improved DeepLabV3+ model demonstrated superior performance, accurately segmenting vegetation classes while excelling in boundary recognition and region delineation. Notably, it achieved precise boundary processing in Masson pine and mixed forest areas, with results closely aligned with ground truth labels. These outcomes underscored its enhanced segmentation capability and robustness.

#### 4.4.3. Model Generalization Ability Verification

**Figure 11:**
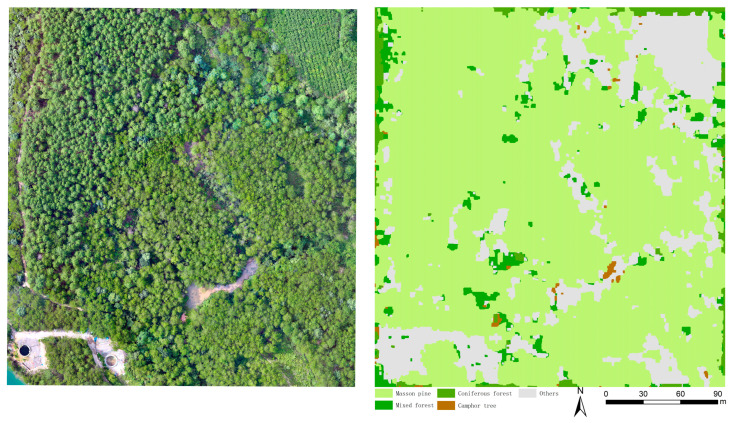
Improved DeepLabV3+ model prediction results.

To further evaluate the robustness and generalization capability of the proposed model across different geographical environments and to address the potential limitations associated with a single study site, a distinct and geographically independent test area was selected for validation experiments, separate from the original 300 m × 300 m study plot. Although this validation area shares a similar tree species composition with the primary experimental site, it exhibits differences in spatial distribution and topographic features, making it an ideal candidate for assessing the model’s predictive capability on unseen data. The model trained in the primary experiment was directly applied to this independent validation dataset without any additional fine-tuning. Visualization of the classification results (Figure 11) indicates that the classification patches generated by the model exhibit remarkable spatial continuity. The model not only accurately reconstructed the dominant spatial distribution patterns of vegetation but also effectively identified tree species with sparse distributions and small sample sizes, successfully overcoming the interference caused by landscape heterogeneity.

Statistical data (Table 6) reveals that the model achieved an Overall Accuracy (OA) of 94.18% and a Kappa coefficient of 0.8762 in the independent test area, demonstrating a high degree of consistency between the predicted results and ground truth. Specifically, the model exhibited exceptional sensitivity and specificity for the dominant species, Masson pine (Producer’s Accuracy: 95%; User’s Accuracy: 98%). Furthermore, even for the “Others” category, which involves a complex background composition, the accuracy remained stable at 92%. These results compellingly demonstrate that the proposed model not only effectively learns local features but also captures universal spectral and textural regularities, thereby possessing reliable cross-regional mapping capabilities.

## 5. Discussion

### 5.1. Determination of Segmentation Scale and Features

In remote sensing image classification, methodologies are broadly categorized into pixel-based (PBIA) and OBIA approaches [46]. Traditional PBIA often suffers from pronounced “salt-and-pepper noise” in high-resolution data due to spectral heterogeneity and inter-class similarity, limiting classification accuracy [47]. In contrast, OBIA improves robustness by segmenting images into homogeneous object units and integrating spectral, textural, geometric, and spatial contextual features [48,49], an advantage validated in tree species classification, both in this study and prior research [10]. However, OBIA performance hinges on segmentation parameter optimization and feature selection. For segmentation, the scale parameter (SP) critically influences accuracy. This study used the ESP2 tool to quantify local variance change rates, objectively determined an optimal SP = 190 by identifying curve peaks (Figure 6), and mitigated the subjectivity of traditional trial-and-error methods. Compared to prior work [50], shape (0.3) and tightness (0.5) factors demonstrated generalized effectiveness, yielding segments with superior canopy edge integrity and internal homogeneity. In feature selection, high-dimensional spaces risk the “curse of dimensionality,” so RFECV was applied. Through 5-fold cross-validation (Figure 8), 18 key features were selected from 86 initial candidates: 7 spectral, 5 vegetation indices, and 6 positional features. This subset retained discriminative information while reducing dimensionality, enhancing classification efficiency over traditional methods. Notably, discrepancies existed with prior literature: Qian et al. [51] reported an optimal SP = 275 for urban trees, likely due to study area differences in species composition and canopy structure. Unlike Wang [1], who relied on random forest importance rankings, this study ensured feature subset stability via RFECV iterative training, directly improving accuracy. These methods offered robust technical support for complex forest scene classification—especially with high-resolution UAV images—and effectively addressed “same object, different spectrum” and “same spectrum, different object” challenges.

### 5.2. Label Optimization by RFVI Method

In deep learning model training, label quality directly impacts classification performance. Traditional manual visual interpretation ensures high accuracy but is inefficient, while purely algorithmic label generation improves speed yet risks elevated misclassification due to spectral similarity and edge mixed-pixels in high-resolution imagery [52]. To resolve this trade-off, we proposed an RFVI method: homogeneous object units from multiscale segmentation, combined with 18 key features selected via RFECV from 86 initial candidates (reduced multicollinearity), leveraged RF’s ensemble learning to boost pre-classification efficiency while maintaining acceptable accuracy [53], addressed traditional manual inefficiency, and mitigated ArcGIS annotation biases. Targeted optimizations addressed three challenging scenarios: edge mixed-pixel regions (corrected via morphological features and spatial context), shaded spectral aberration areas (aided by multi-temporal data), and young tree-shrub morphological similarity (distinguished using textural and geometric features). Professional interpreters refined these critical areas with field data, modifying approximately 15% of the initial predictions to correct misclassifications and boundary errors. This yielded final labels with 98.55% OA and 97.98% Kappa (Table 3), closely aligning with ground truth (Figure 9). This method resolved the “efficiency-accuracy” dilemma in label production and provided a reusable framework for intelligent forestry remote sensing annotation.

### 5.3. Performance Analysis of the Improved DeepLabV3+ Model

The traditional DeepLabV3+ model employs Xception as its backbone. Although the ASPP module enhances multiscale feature extraction [38,54,55], its high parameter count limits deployment on resource-constrained devices. Additionally, tree class boundaries in remote sensing images often exhibit ragged edges due to spectral similarity—particularly in complex vegetation cover—a limitation stemming from the original model’s insufficient lightweight design and poor local detail sensitivity. To address these challenges, we proposed an improved scheme that integrated a lightweight backbone and dual attention mechanisms. Replaced Xception with MobileNetV2 reduced parameters to 5.81 M (78.7% reduction), though initial testing showed OA and Kappa declines to 88.73% and 84.31%, respectively. This performance drop can be attributed to the inherent trade-off in lightweight architectures. As noted by Mark et al. [56], the depthwise separable convolutions in MobileNetV2, while efficient, possess lower representational capacity compared to the standard convolutions in Xception. This results in a loss of fine-grained spatial details and global contextual information. To compensate, we innovatively embed CBAM_1_ (encoder) and CBAM_2_ (decoder) to form a dual-attention optimization mechanism. Adding CBAM_1_ to MobileNetV2 rebounded OA to 90.73% (2% improvement). Crucially, the attention mechanism acts as a feature recalibration tool: the CAM suppresses redundant channels that carry noise, while the SAM enhances sensitivity to spatial structures like tree boundaries, thus mitigating lightweight backbone degradation. This aligned with Chen et al.’s [54] “attention compensation mechanism,” where dynamic feature weighting balanced efficiency and accuracy. CBAM_2_, further embedded in the decoder, boosted OA and Kappa to 94.91% and 92.89% with only a 0.10 M parameter increase, indicating that CBAM_2_ optimized shallow feature boundaries via spatial attention while preserving a lightweight design. Given the significant spatiotemporal heterogeneity inherent in forest remote sensing imagery, we further validated the model’s generalization capability on a completely independent test site. The results demonstrated that the improved model maintained a stable OA of 94.18%, significantly outperforming the original DeepLabV3+ under identical conditions. This robustness is attributed to the parameter sparsity of MobileNetV2, which effectively reduces the risk of overfitting, combined with the CBAM modules that focus on key semantic features, thereby enhancing the model’s resistance to environmental noise.

In summary, the improved model not only achieves a balance between high precision (94.91%) and lightweight design but also demonstrates the robustness required for complex forestry scenarios. By successfully navigating the efficiency–accuracy trade-off, this approach provides a feasible methodological framework for precise monitoring of highly heterogeneous forest areas.

### 5.4. Shortcomings and Prospects

The primary objective of this study was to improve the DeepLabV3+ model for enhanced training efficiency and tree species classification accuracy. While we have validated the model’s performance on an additional study area to assess its generalization capability, certain limitations remain. First, the current validation is based on UAV images acquired in specific seasons. Seasonal variations, such as autumnal leaf senescence and winter defoliation, may affect spectral features, potentially reducing segmentation consistency if the model is applied to year-round monitoring without adaptation. Additionally, although the model performed well on UAV data, its applicability to broader scales, such as satellite imagery with lower spatial resolution, remains to be fully explored. Second, regarding comparative analysis, this study primarily focused on benchmarking the improved model against the original architecture and traditional heavyweight models (e.g., U-Net, PSPNet) to highlight the efficiency gains from our structural optimizations. A comprehensive comparison with other state-of-the-art lightweight semantic segmentation networks, such as SegNet, ENet, or FastSCNN, was not included within the scope of this work. We acknowledge that such a comparison would provide valuable insights into the relative competitiveness of our approach within the broader landscape of lightweight architectures.

To address these limitations, future work will focus on (1) extending the dataset to include multi-seasonal and multi-source images (including satellite data) to further test robustness in diverse environmental conditions and (2) conducting rigorous benchmarking against a wider range of lightweight algorithms to further evaluate and optimize the trade-off between model size, segmentation speed, and accuracy.

## 6. Conclusions

In this study, we developed a DeepLabV3+ model that integrated OBIA and a lightweight attention mechanism, enabling accurate tree species classification in complex forest scenarios. This method utilized high-resolution remote sensing images derived from UAV and employed the RFVI method to achieve effective label refinement, resulting in the following key findings:

(1) Through integrated analysis of multiscale segmentation algorithms and RFECV, optimal OBIA scale parameters and feature combinations were determined, substantially mitigating the impact of manual intervention on classification outcomes.
(2) The RFVI method was developed to optimize labels, enhancing both production efficiency and annotation quality. This approach established an efficient and reliable label optimization pipeline, providing a high-quality data foundation for deep learning model training.
(3) The model achieved breakthrough performance via lightweight design and enhanced attention mechanisms. The improved model attained an OA of 94.91% and a Kappa of 92.89%, representing increases of 2.91% and 4.11% over the original DeepLabV3+. Compared to U-Net and PSPNet, an OA improved by 2.0% and 4.55%, respectively, while model parameters were drastically reduced to 5.91 M (a 78.7% decrease). This demonstrated a high-precision, efficient, lightweight solution for UAV-based tree species segmentation in complex forest images.

## Figures and Tables

**Table 1:** Characteristic variables [32].

Feature Type	Description
Spectral features	Mean value of each band, Standard deviation, Brightness, Max. diff
Vegetation indices	Blue, Green, Red, EXG, NGBDI, NGRDI, RGRI, GLI
Geometric features	Area, Length/Width, Length, Width, Border length, Shape index, Density, Compactness, Asymmetry, Elliptic fit, Rectangular fit, Main direction
Positional features	X Center, X Max, X Min, Y Center, Y Max, Y Min, Z Center, Z Max, Z Min, Time, Time Max, Time Min, Distance to scene border
Textural features	GLCM Mean, GLCM Entropy, GLCM Homogeneity, GLCM Contrast, GLCM Dissimilarity, GLCM Correlation, GLDV Entropy, GLDV Mean, GLDV Contrast

**Table 2:** Calculation formula for the vegetation index [32].

Vegetation Indices	Calculation Formula
Blue	B/(R + G + B)
Green	G/(R + G + B)
Red	R/(R + G + B)
EXG	2 × G − R − B
NGBDI	(G − B)/(G + B)
NGRDI	(G − R)/(G + R)
RGRI	R/G
GLI	(2 × G − R − B)/(2 × G + R + B)

**Table 3:** Result accuracy verification table.

Accuracy Verification	Masson Pine	Mixed Forest	Others	Camphor Tree	Coniferous Forest
PA	0.97	1	1	1	1
UA	1	0.98	0.96	0.99	0.98
OA	0.9855				
Kappa	0.9798				

**Table 4:** The impact of improved modules on model performance.

Original Model	MobileNetV2	CBAM_1_	CBAM_2_	OA/%	Kappa/%	Model Parameters/M
✓				92	88.78	27.30
✓	✓			88.73	84.31	5.81
✓	✓	✓		90.73	87.01	5.90
✓	✓	✓	✓	94.91	92.89	5.91

Note: The “✓“ in the table indicates that the corresponding module is integrated into the model.

**Table 5:** Experimental results of different semantic segmentation models.

Semantic Segmentation Model	OA/%	Kappa/%	Model Parameters/M	PA/%				
				Masson Pine	Mixed Forest	Others	Camphor Tree	Coniferous Forest
PSPNet	90.36	86.54	47.76	96.34	81.63	83.70	88.89	92.86
DeepLabV3+	92.00	88.78	27.30	96.75	84.69	88.04	88.89	95.24
U-Net	92.91	90.12	31.04	95.12	93.88	89.13	88.89	92.86
Improved DeepLabV3+	94.91	92.89	5.91	97.56	93.88	90.22	93.06	95.24

**Table 6:** Improved DeepLabV3+ model prediction accuracy.

Accuracy Verification	Masson Pine	Mixed Forest	Others	Camphor Tree	Coniferous Forest
PA	0.95	0.92	0.92	0.75	0.92
UA	0.98	0.76	0.91	0.60	0.79
OA	0.9418				
Kappa	0.8762				

## Data Availability

Code and datasets may be available upon request.
